# Conjugation of a *Cryptococcus neoformans*-derived metalloprotease to antifungal-loaded PLGA nanoparticles treats neural cryptococcosis in an *in vitro* model

**DOI:** 10.1371/journal.pone.0340202

**Published:** 2026-01-16

**Authors:** Dylan M. Lanser, Adam Turner, Noah Pacifici, Kiem Vu, Jamal Lewis, Angie Gelli

**Affiliations:** 1 Department of Pharmacology, School of Medicine, University of California, Davis, California, United States of America; 2 Department of Biomedical Engineering, University of California, Davis, California, United States of America; 3 J. Crayton Pruitt Family Department of Biomedical Engineering, University of Florida, Gainesville, Florida, United States of America; Duke University School of Medicine, UNITED STATES OF AMERICA

## Abstract

Overcoming the blood-brain barrier to deliver therapeutics is a major hurdle in treating diseases of the central nervous system. We engineered 4-arm carboxyl terminated poly(D,L-lactide-co-glycolide) nanoparticles with the fungal metalloprotease Mpr1, an enzyme utilized by the neurotropic pathogen *Cryptococcus neoformans* (*Cn*) to cross the blood-brain barrier. Nanoparticles were prepared using a modified single emulsion solvent evaporation technique, and characterized in terms of shape, size, zeta potential, encapsulation efficiency, and toxicity to brain microvascular endothelial cells. Mpr1-functionalized nanoparticles had increased penetration over non-functionalized nanoparticles in an *in vitro* model of the blood-brain barrier. When encapsulating amphotericin B, a potent antifungal drug, Mpr1-functionalized nanoparticles reduced fungal burden in an *in vitro* model of neural cryptococcosis. Loaded nanoparticles also had an 8-fold lower minimum inhibitory concentration against *Cn* and *Candida albicans* (*Ca*) compared to unencapsulated amphotericin B. Results indicate that Mpr1-coating of polymeric nanoparticles is a promising strategy to enhance drug delivery to the brain.

## Introduction

The central nervous system (CNS) is protected by the blood-brain barrier (BBB), which is primarily a product of brain microvascular endothelial cells (BMECs), comprising the brain microvasculature, with support from perivascular astrocytes and pericytes [1]. Tight junctions (TJ) prevent paracellular exchange between the blood and brain. Polarization in the expression of TJ proteins and efflux pumps effectively excludes the vast majority of pathogens and toxins from entering the CNS, but also excludes many critical therapeutics, [2] posing a problem when a pathogen becomes established behind the BBB. The treatment of malignant and neurological conditions can similarly be inhibited by the BBB. However, neurotropic pathogens may also serve as a source of inspiration for developing strategies to improve CNS-targeting therapies.

The neurotropic fungal pathogen *Cn* is one such organism. *Cn* utilizes several mechanisms to invade the CNS including a transcellular route in which *Cn* is endocytosed from microvascular circulation by brain endothelial cells and subsequently exocytosed into the perivascular space and eventually to the brain parenchyma [3,4]. Although CNS infection by *Cn* is fatal if untreated, resulting in greater than 100,000 deaths annually, [5] transcellular BBB crossing itself may not break down the BBB, [6] suggesting that the pathway utilized by *Cn* to traverse the BBB can be safely repurposed. *Cn* secrets a metalloprotease, Mpr1, that is both necessary [7] and sufficient [8] for BBB crossing, and can enhance BBB-crossing of quantum-dot nanoparticles [9]. This fungal-derived metalloprotease may therefore facilitate CNS drug delivery. In this study, we evaluated Mpr1 compatibility with polymeric (PLGA) nanoparticles encapsulating an antifungal drug, amphotericin B (AmB), for delivery across the BBB and treatment of neural cryptococcosis.

## Materials and methods

### Chemicals and strains

All poly(lactic-co-glycolic) acid polymers, including 4-arm PLGA 20 kDa and PLGA-Fluorescein (PLGA-FITC) 20 kDa, were purchased from Nanosoft Polymers (Lewisville, North Carolina). Amphotericin B (AmB), dichloromethane (DCM) and dimethyl sulfoxide (DMSO) were obtained from Millipore Sigma (Burlington, MA). 1-ethyl-3-(3-dimethylaminopropyl)carbodiimide hydrochloride (EDC), N-hydroxy succinimide (NHS), and *Pichia pastoris* GS115 were purchased from ThermoFisher Scientific (Waltham, MA, USA). KN99, a common *Cn* serotype A laboratory strain derived from H99 was used for antifungal testing [10]. *Ca* isolates were provided by Dr. G.R. Thompson, University of California, Davis. Drug-resistance of isolates was confirmed by the Fungus Testing Laboratory (San Antonio, TX) and provided by Dr. Thompson. Strains were recovered from −80°C frozen stocks, grown in YPD (1% yeast extract, 2% bacto-peptone, and 2% dextrose) at 30°C. The human brain endothelial cell line (hCMEC/D3) was a gift from Dr. Babette Weksler (Cornell University), who developed and characterized it [11].

### Mpr1 recombinant protein production

Mpr1 was produced as described previously with some modifications [9,12]. Briefly, *Pichia pastoris* expressing *Cn* Mpr1-HIS-tagged under the *AOX1* methanol-induced promoter was grown over 24 h in complex media at 30°C. Cultures were then changed into media containing 0.5% v/v methanol. After 24 h, 0.5% w/v sorbitol was added to the culture, followed by a further 24 h incubation. Mpr1 was precipitated from the supernatant with HIS-affinity magnetic beads (PureCube INDIGO Ni-MagBeads, Cube Biotech). Beads binding Mpr1 were washed with 50mM NaH_2_PO_4_, 300 mM NaCl, and 20 mM imidazole pH 8.0, then eluted with 50mM NaH_2_PO_4_, 300 mM NaCl, and 500 mM imidazole pH 8.0. Following concentrating in a 10 kDa centrifugal filter unit (4000 rcf, 15 min, 4°C) (Amicon), protein concentration was determined by Bradford assay. Mpr1 was either used immediately or flash-frozen in liquid nitrogen and stored at −80°C [12].

### Preparation of nanoparticles

We modified a previously described oil-in-water single emulsion technique to prepare 4-arm PLGA nanoparticles (4PNPs) [13]. PLGA was dissolved in DCM at a concentration of 100 mg/mL. For some experiments using fluorescence as a readout, nanoparticles were fabricated from a 1:1 w/w mixture of 4-arm PLGA and PLGA-FITC. For experiments involving loaded AmB nanoparticles (4PANPs), 10 mg AmB were dissolved in 0.5 mL 100% DMSO, then added dropwise to the PLGA-DCM solution for a final 2% w/w ratio of AmB. The dissolved polymer was then added dropwise to a 5% w/v of polyvinyl alcohol (PVA) aqueous mixture. Emulsification took place under ice bath sonication (Branson Sonicator 450V) at 60% amplitude for 5 min. This emulsion was then added dropwise into a 2.5% w/v PVA aqueous mixture. The mixture was left to stir at 600 rpm, room temperature overnight, allowing the organic phase to evaporate. Particles were pelleted at 20,000 x g for 20 min and washed twice with ultrapure deionized water, then lyophilized for 48 h. Mpr1 was conjugated to 4PNPs (Mpr1–4PNPs) by initially mixing particles, EDC, and NHS in a 1:1:2 w/w ratio in 1 mL PBS for 30 min at 4°C under constant rotation, and further washed with PBS to remove excess N-hydroxysulfosuccinimide and remaining EDC. Following washes, 2 mg of Mpr1 was added for a further 4 h incubation at 4°C to covalently conjugate Mpr1 to the nanoparticles via the primary amine-reactive NHS site. In some preparations of AmB PNPs intended for MIC determination, this conjugation step was extended to 24 hours to determine whether prolonged incubation diminished subsequent antifungal activity. Mpr1–4PNPs were centrifuged at 10,000 rcf, washed twice in PBS with 1% bovine serum albumin, further washed with ultra-pure deionized water, and resuspended in 500 µL PBS (2 mg/mL 4PNPs). 4PNPs were either used immediately or flash-frozen and stored at −80°C. Control nanoparticles were fabricated identically, without the introduction of Mpr1.

### Susceptibility testing of antifungal activities of nanoparticle formulations

Susceptibility testing was carried out in RPMI 1640 medium containing L-glutamine, without sodium bicarbonate, with MOPS, pH 7.0 in flat-bottom 96-well plates (Costar). Inoculums of (*Cn*)and (*Ca*) were prepared in accordance with the CLSI standard (M27-A3), added to the 96-well plates containing two-fold serial dilutions of nanoparticles and incubated for 48 h at 35°C without shaking. Readings were taken by visual inspection of the opacity of the wells. The MIC of nanoparticles was defined as the lowest drug concentration in a well at which 100% reduction in optical density was observed compared to the untreated control.

### Amphotericin B entrapment efficiency

4PANPs were dissolved with 5 mL DMSO to a final concentration of 0.8 mg/mL and placed in dialysis tubing and the tubing was then placed in 100 mL PBS. After 48 h, the dialysate was collected and centrifuged at 20,000 rcf. The absorbance of the supernatant was measured at 416 nm and compared to an AmB standard curve. The entrapment efficiency (EE%) was calculated using the following equation.


$$EE\%=\frac{SupernatantA\text{4}\text{1}\text{6}}{Theoretical\text{1}\text{0}\text{0}\%A\text{4}\text{1}\text{6}}*\text{1}\text{0}\text{0}$$

### Proteolytic activity of Mpr1–4-arm-PLGA nanoparticles (Mpr1–4PNPs)

The proteolytic activity of Mpr1 was determined before and after bioconjugation to 4PNPs using a FRET-based protease activity assay (Pierce Protease Activity Assay Kit, ThermoFisher Scientific). Nanoparticles were diluted after conjugation (real and mock) to Mpr1. Fluorescently labeled casein served as a generic proteolytic substrate. Proteolytic activity, measured by an increase in relative fluorescence units (RFU) as FRET quenching decreased upon substrate degradation, was measured over 65 min in an automated plate reader (SpectraMax M5, Molecular Devices, San Jose, CA). Trypsin served as a positive control for proteolytic activity.

### Physical characterization of nanoparticles

Characterization of nanoparticles was performed by scanning electron microscopy (SEM) and with a dynamic light scattering (DLS)/zeta potential analyzer (Zetasizer, Malvern Instruments). Zeta potential and DLS measurements were performed with lyophilized PNPs dispersed in 1mL of PBS.

### Cell viability assay

Cytotoxicity of PNPs was determined *in vitro* on hCMEC/D3 cells using an MTT viability assay (MTT Assay Kit, Abcam, Cell Biolabs, San Diego, CA). Briefly, hCMEC/D3 cells were grown to confluence in a 96-well, flat-bottom plate. Cells were then treated with Mpr1, Mpr1–4PNPs, control 4PNPs, or PBS, and incubated at 37°C, 5% CO_2_ for 24 h. Dilutions of PNP treatments (Mpr1 and control) were made at the end of preparations, with dilutions being made on the basis of the starting PLGA mass, allowing for normalization of the number of particles between Mpr1 PNPs and control PNP dilutions. Cytotoxicity was determined via the concentration of formazan in each well by measuring absorbance at 590 nm, which is positively correlated with cell viability. Cytotoxicity was calculated as the percent difference between the absorbance at 590 nm compared to control wells.

### Blood-brain barrier penetration fluorescence-based assay

Immortalized brain microvascular endothelial cells (hCMEC/D3 cell line, up to passage 31) were seeded in 24-well transwell plates (8 µm pore diameter, Corning) at 50% confluent density in 1X EGM-2 media (Lonza, Vacaville, CA) supplemented with 2.5% fetal bovine serum (FBS, Gibco) and 1 ng/mL recombinant human fibroblast growth factor (hFGF, Gibco, ThermoFisher). After reaching confluence (5 d), cells were differentiated over 2 d in first 50% then 25% of the original FBS and hFGF concentrations. Nanoparticles (20 µg PLGA/mL) were added to 8 wells per treatment. After 4 h, media was removed from both above and below the inserts and relative fluorescence units (RFU) were measured on a plate reader (excitation 495 nm, emission 519 nm). Background from control wells was subtracted from raw readings. The ratio of fluorescence in the bottom of each well over the top was used as a measure of PNP crossing.

### Antifungal activity of Mpr1–4PANPs in BBB transwell model

Nanoparticles were diluted 1:500 (4 µg/mL PLGA, 80 ng/mL AmB in loaded PNP treatments) in the top chamber and 2.4x10^4^ *Cn* H99 cells were added to the bottom. After 4 h incubation at 37°C, and 5% CO_2_, inserts containing hCMEC/D3 cells, along with any PNPs that had not crossed, were removed. The plate was returned to the incubator for a further 20 h, after which 100 µL were removed for plating on SBD agar. Colony forming units (CFU) were determined after 48 h incubation at 30ºC.

### Statistical analysis

Results are expressed as Mean + /- standard deviation. Statistical analysis was performed in R v.4.2.2 (R Core Team, 2022) or with GraphPad Prism 10. All two-way comparisons are two-sided Welch Two Sample *t*-tests.

## Results

### Physicochemical characteristics of modified polymeric PLGA nanoparticles

**Fig 1:**
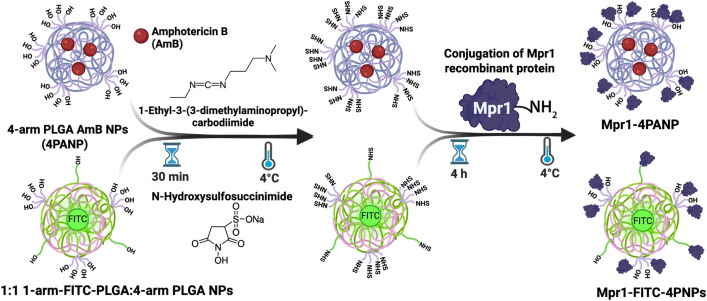
Schematic representation of the methodology used to generate PLGA-based nanoparticles (PNPs) conjugated to Mpr1 recombinant protein. Top panel: Mpr1-AmB-loaded-PNPs (Mpr1-4PANPs); Bottom panel: Mpr1-FITC-PNPs (Mpr1-FITC-4PNPs).

We adapted existing nanofabrication processes to produce PLGA nanoparticles (PNPs) (Fig 1)

**Fig 2:**
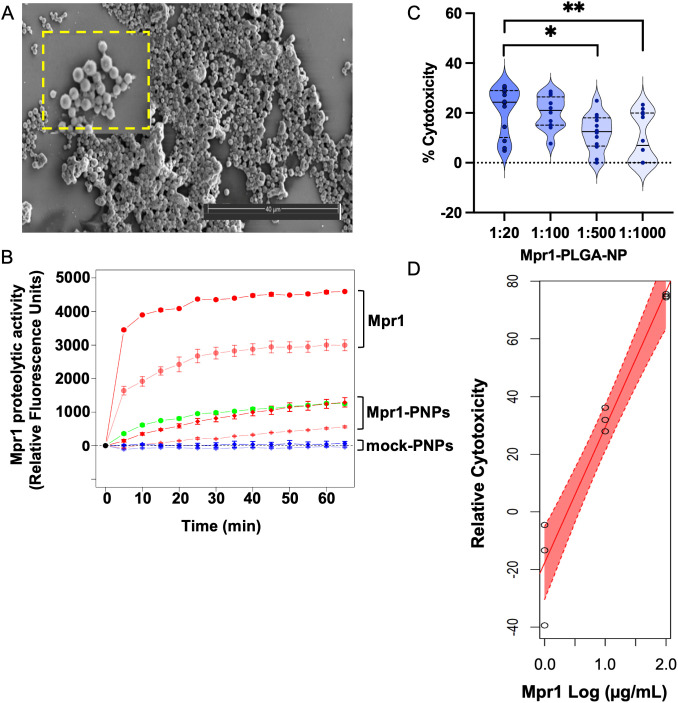
4-arm PLGA nanoparticles (PNPs) conjugated to Mpr1 retain proteolytic activity and show modest cytotoxicity. **(A)** Scanning electron micrograph (SEM) of 4-arm PLGA nanoparticles (4PNPs). Scale = 40 µm. **(B)** Proteolytic activity of Mpr1 and Mpr1–4arm PLGA NPs (Mpr1–4PNPs). Points represent the average reading of three triplicate wells, normalized to the average fluorescence from tris-buffered saline (TBS) control wells. Error bars represent + /- SD. Mpr1 (100 µg/mL, dark red circles, 10 µg/mL, light red circles); Mpr1 nanoparticles at 4 mg/mL PLGA (dark red diamonds) and 400 µg/mL PLGA (light red diamonds); blank nanoparticles at 4 µg/mL PLGA (dark blue diamonds, and 400 µg/mL PLGA (light blue diamonds); and trypsin control (0.5 µg/mL, green). Proteolytic activity expressed as RFUs (relative fluorescence units; Excitation 485 nm, Emission 535 nm). Experiment was repeated six times. **(C)** Assessment of dose-dependent cytotoxicity of Mpr1 on human brain endothelial cells (hCMEC/D3 cell line) by MTT assay. Violin plots of Mpr1-functionalized PLGA nanoparticles (Mpr1–4PNPs) show relatively modest cytotoxicity at higher dilutions. Dilutions were performed on nanoparticles after normal conjugation. **(D)** Dissolved Mpr1 with cytotoxicity measured relative to buffer. The shaded region in (D) represents 95% CI (Confidence Interval).

[14–16]. In order to maximize the number of surface carboxylic groups available for bioconjugation, we opted for the 4-arm PNPs (4PNPs). Some 4PNPs were loaded with Amphotericin B (AmB, PANPs), a polyene antifungal drug with excellent clinical efficacy for systemic mycoses, low rates of resistance and broad-spectrum activity [17]. We determined that the encapsulation efficiency of AmB within 4PNPs was 68.2%, and 55.1% within 1PNPs. Scanning electron micrographs of 4PANPs showed particles that were spherical and homogenous in shape and size (Fig 2A). Single-arm AmB-loaded PNPs (1PANPs) were similar to 4PANPs (Table 1).

**Table 1:** Physical characteristics of nanoparticle formulations.

	FITC-1PNPs	4PANPs	1PANPs
PDI	0.26	0.18	0.08
Hydrodynamic Diameter (nm)	458 ± 88.1	212.3 ± 78.9	207.9 ± 60.55
Zeta Potential (mV)	−3.93	−2.85	−2.65

Measured zeta potential, polydispersity index (PDI) and mean size of each formulation.

### Proteolytic activity of loaded-PLGA nanoparticles conjugated to recombinant Mpr1

We then conjugated cryptococcal recombinant Mpr1 protein to 4PNPs (Mpr1–4PNPs). Large scale production of recombinant Mpr1 with high purity was achieved by expression of *MPR1* in *Pichia pastoris,* a non-pathogenic yeast [9,12]. We determined that proteolytic activity of recombinant Mpr1 was retained on 4PNPs (Fig 2B). In contrast, equivalent concentrations of 4PNPs which were processed identically to Mpr1–4PNPs, but lacked Mpr1, had no detectable proteolytic activity (Fig 2B).

We found that Mpr1–4PNPs displayed modest cytotoxicity when compared to non-functionalized 4PNPs, and that free Mpr1 concentration was positively correlated with cytotoxicity relative to buffer control (*P* < 0.001) (Fig 2C and 2D). A reduction in brain endothelial cell viability was observed only at lower dilutions of Mpr1–4PNPs (*P* = 0.015 at 1:20 or 0.1 mg/mL; and *P* = 0.003 at 1:100 dilution or 20 µg/mL) in contrast to higher dilutions of 1:500 and 1:1000 where the level of cytotoxicity was not significantly different from the baseline (Fig 2C). Based on these results, *in vitro* assays of Mpr1–4PANP fungicidal activity were performed at high (1:500) PNP dilutions, reducing the likelihood that Mpr1-associated cytotoxicity contributed to increased PNP crossing.

### Antifungal activity and off-target effects of AmB-encapsulated, Mpr1-functionalized polymeric nanoparticles

AmB-encapsulated Mpr1–4PNPs (i.e., Mpr1–4PANPs) and 4PANPs were examined for antifungal activity against *Cn* and *Ca*, with non-encapsulated AmB and non-conjugated PNPs as control treatments (Table 2). Antifungal susceptibility assays showed no growth inhibition among fungi treated with non-loaded PNPs. In contrast, inhibition of both fungal strains was observed with 1.3 μg/mL of free, unencapsulated AmB (Table 2). All PANPs, including Mpr1–4PANPs, inhibited fungal growth of both strains at a lower equivalent concentration of AmB compared to non-encapsulated AmB. An 8-fold decrease in MIC for 4PANPs against *Cn* and *Ca* was observed relative to non-encapsulated AmB, indicating that encapsulation promoted greater antifungal activity of AmB (Table 2). Our results suggest that conjugation of active Mpr1–4PANPs did not inhibit antifungal activity of AmB.

**Table 2:** Susceptibility of *Cryptococcus neoformans* and *Candida albicans* to antifungal activity of various nanoparticle formulations, including AmB-loaded and Mpr1-conjugated (with 4- or 24-hour conjugation reaction) PLGA nanoparticles.

	*Cryptococcus neoformans*	*Candida albicans*
**Formulations**	^1^ **MIC (µg/mL)**	^1^ **MIC (µg/mL)**
Non-encapsulated AmB	1.3	1.3
1-arm PLGA nanoparticles (1PNPs)	No activity	No activity
4-arm PLGA nanoparticles (4PNPs)	No activity	No activity
AmB-loaded 1-arm PLGA nanoparticles (1PANPs)	0.060	0.080
AmB-loaded 4-arm PLGA nanoparticles (4PANPs)	0.120	0.160
AmB-loaded 4-arm PLGA nanoparticles conjugation control (4 h) (4PANPs)	0.244	0.325
AmB-loaded 4-arm PLGA nanoparticles conjugation control (24 h) (4PANPs)	0.163	0.325
AmB-loaded 4-arm PLGA nanoparticles + Mpr1 (4h) (Mpr1–4PANPs)	0.163	0.163
AmB loaded 4-arm PLGA nanoparticles + Mpr1 (24h) (Mpr1–4PANPs)	0.163	0.325

^1^MIC, minimal inhibitory concentration; AmB – amphotericin B; Mpr1- recombinant secreted metalloprotease; PLGA – poly(lactic-co-glycolic acid).

### BBB crossing and antifungal effects of Mpr1-functionalized AmB-loaded polymeric nanoparticles in a transwell BBB model simulating treatment of CNS-disseminated cryptococcosis

**Fig 3:**
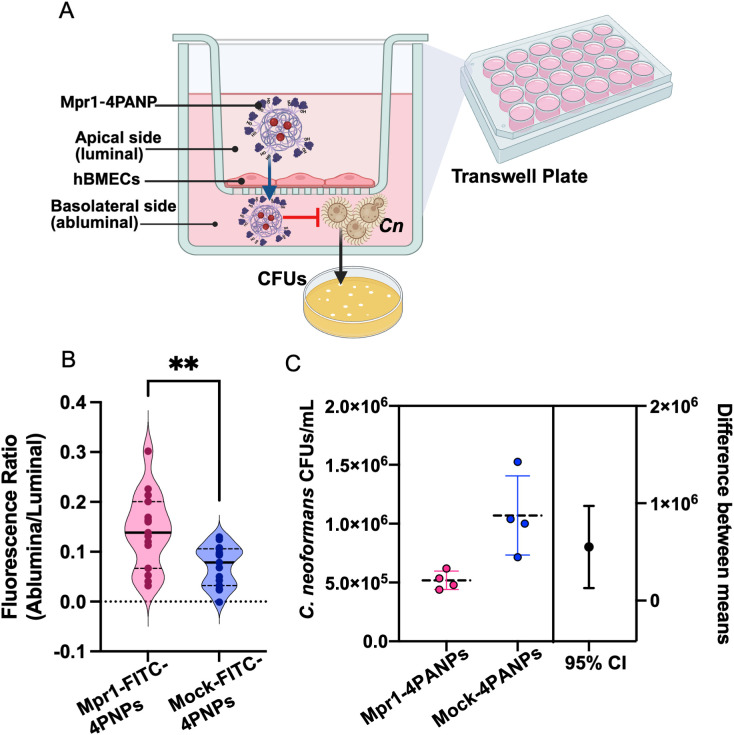
Mpr1 promotes payload (AmB) delivery across the blood-brain barrier. **(A)** Schematic of a modified 2D *in vitro* transwell model of the blood-brain barrier (BBB) with hCMECs to simulate treatment of CNS-disseminated cryptococcosis. Fungal cells were seeded on the bottom-well below the transwell (‘brain’ or abluminal side) and nanoparticles were added to media above the transwell (‘blood’ or luminal side). **(B)** Plot of the transcytosis of Mpr1-4ARM-PLGA-FITC (Mpr1-FITC-4PNPs) nanoparticles in a transwell-based *in vitro* BBB model. Mpr1-FITC-4PNPs and mock FITC-4PNPs were introduced at 40 µg/mL in the apical (luminal) compartment. The ratio of fluorescent signal in the basolateral (abluminal) over the apical compartment after 12 h was used as an indication of crossing (two-tailed t-test **P = 0.005). Bars represent mean + /- SD. The shape (circle, square, triangle) of each data point on the graph corresponds to a replicate. Assay was repeated three times. **(C)** Fungicidal activity of amphotericin B-loaded Mpr1-4ARM-PLGA (Mpr1-4PANPs) nanoparticles in the transwell-based *in vitro* BBB model. Scatter plot (mean and SD, *P =* 0.018) of colony forming units (CFUs) collected from the well below the transwell (basolateral). The 95% CI around the difference in means is depicted. Assay was repeated twice.

To determine whether Mpr1 could support the delivery of a payload across the BBB, we used a transwell-based *in vitro* BBB model with immortalized human brain endothelial cells (hCMEC/D3) [11,18–20]. In this model, hCMEC/D3 cells were grown on a semi-permeable membrane, with the well contents beneath representing the brain side of the BBB and the volume above representing the blood side (Fig 3A).

PNPs made from a 1:1 ratio of 1-arm-PLGA-FITC and 4-arm-PLGA-Mpr1 (Mpr1-FITC-4PNPs), along with mock-conjugated controls (FITC-4PNPs) were generated and tested *in vitro*. The ratio of fluorescence between the bottom (‘brain’) and top (‘blood’) compartment was significantly higher for Mpr1-FITC-4PNPs than FITC-4PNPs (*P* = 0.005), indicating that a greater number of Mpr1-functionalized 4PNPs crossed the BBB compared to non-functionalized 4PNPs (Fig 3B).

We used a modified version of the transwell BBB model to simulate treatment of CNS-disseminated cryptococcosis. In this model, *Cn* were pre-seeded on the bottom (‘brain’) chamber of the transwell prior to addition of Mpr1–4PANPs or 4PANPs to the top (‘blood’) chamber. *Cn* CFUs recovered from the ‘brain’ side were approximately two-fold lower after 24 h incubation with Mpr1–4PANPs compared to incubation with non-functionalized 4PANPs (Fig 3C and 3D). These results are consistent with Mpr1’s ability to facilitate crossing of the BBB and delivering a therapeutic.

## Discussion

PLGA is a biocompatible, biodegradable polymer well-profiled in terms of effects on drug potency, release profiles and loading potential [2,21]. These properties have resulted in PLGA-based delivery systems being approved by the US FDA [22]. Simultaneously, nano formulation of drugs such as AmB have been demonstrated to reduce off-target effects through improved targeting [17]. Both diameter and zeta potential can impact crossing of the BBB and diffusion through the brain interstitial space [23]. Generally, PLGA nanoparticles have negative surface charges but can become neutral or positive with surface modification. The zeta potentials of our nanoparticles were close to neutral, which is favorable to passage across the BBB [24] and low cytotoxicity [25]. Although our nanoparticles varied in size when loaded with AmB, functionalized nanoparticles with a wide range of diameters have been shown to cross the BBB [23]. Notably, synergy between PLGA and AmB has been previously observed [18,24,26]. The enhanced antifungal activity of encapsulated AmB may be due to the affinity of PLGA nanoparticles for the fungal cell wall [24], which would support the release of AmB proximal to its target (ergosterol) in the fungal membrane [27], resulting in fungal cell death [17].

While this work supports previous studies demonstrating that Mpr1 is sufficient to promote BBB crossing [9,28], the underlying mechanism is largely undefined. Because elimination of Mpr1 proteolytic activity was found to prevent BBB crossing, [8] an Mpr1-cleavable substrate expressed on the brain endothelium may facilitate transcellular crossing of *Cn* [4]. In the current study, Mpr1 conjugated to 4PNPs retained proteolytic activity and were therefore capable of acting on Mpr1substrate(s). Mpr1-coated quantum dots were shown to induce endocytic pits on the surface of brain endothelial cells [9], and Mpr1-expressing *Cn* appeared to activate an endocytic process on the surface of these cells [3,5,9]. Therefore, Mpr1–4PNPs likely crossed brain endothelial cells by inducing endocytic vesicle formation. Characterization of the Mpr1 substrate(s) may allow for further optimization of Mpr1 for BBB crossing [4].

Several other proteins have been considered for functionalizing nanoparticles for trans-BBB drug delivery, including transferrin [29,30] and bovine serum albumin (BSA) [31,32] Cationic albumin has been shown to cross the BBB on its own by adsorptive transcytosis [33] We compared particles conjugated to BSA and Mpr1 (S1 Fig), however, like many exogenous proteins, albumin infiltration of the brain parenchyma brain is known to activate astrocytes and microglia [34] (thus presenting its own set of drawbacks as a nanoparticle conjugate. Also, since it is difficult to equalize the amount of distinct proteins conjugated onto nanoparticles, comparisons are inherently difficult.. The use of a catalytically inactivate version of Mpr1 to determine whether proteolytic activity is indeed necessary for enhanced BBB crossing of PLGA nanoparticles, as has been determined in other contexts [8], would be informative.

Although we observed a trend towards Mpr1-associated cytotoxicity at the nanoparticle working concentration, the reduction in fungal CFU we observed in our BBB model is proportionally greater, suggesting that crossing occurred mainly through a cell-mediated interaction rather than the toxic effect of Mpr1. Additionally, cytotoxicity was measured after a longer incubation period than was used in the *in vitro* BBB model, and therefore represents an upper bound on the toxicity of Mpr1–4PNPs in that model. However, it is likely that the Mpr1-PANPs we describe would be favored only in the treatment of already disseminated infections, both due to cytotoxicity concerns and the somewhat lower inhibitory concentrations we achieved with non-conjugated PNPs, and that other PANP configurations would be favored in non-disseminated infections. Reduction of off-target effects through nanoencapsulation of compounds such as AmB may entirely offset Mpr1 toxicity. In animal studies, AmB-loaded PLGA nanoparticles reduced undesirable effects of treatment for paracoccidioidomycosis relative to free AmB [18]. Additionally, in the case of cryptococcosis treatment, patients would have been exposed to Mpr1 from the pathogen. Any potential harm caused by Mpr1 itself must also be judged relative to invasive approaches that have been explored to enhance delivery of therapeutics across the BBB, such as ultrasound, osmotic shock, intracerebroventricular and intrathecal infusion [35].

Finally, our results are indicative of a noninvasive strategy for drug delivery to the CNS. Advances in protein engineering may offer the potential to further increase Mpr1-enhanced CNS entry while simultaneously reducing off target effects [36].

## Supporting information

S1 Fig Comparison of fluorescence signal (RFU) from Mpri-4arm and BSA-4arm FITC-PLGA nanoparticles in the bottom chamber of the transwell-based BBB model after 4 h. Each point represents one well, spread across three independent replicates. Readings are normalized to blank nanoparticle controls run in each replicate and corrected for the baseline fluorescence of the two nanoparticle types, such that the y-axis represents multiples of the blank nanoparticle fluorescence reading after 4 h. The difference between the groups is marginally non-significant (P = 0.064), two-sample t-test with Welch’s correction (t = 2.07, df = 10.38).
(TIFF)

## References

[1] Keaney J, Campbell M. The dynamic blood-brain barrier. FEBS J. 2015;282(21):4067–79. doi: 10.1111/febs.13412 26277326

[2] Pardridge WM. A historical review of brain drug delivery. Pharmaceutics. 2022;14(6):1283. doi: 10.3390/pharmaceutics14061283 35745855 PMC9229021

[3] Chang YC, Stins MF, McCaffery MJ, Miller GF, Pare DR, Dam T, et al. Cryptococcal yeast cells invade the central nervous system via transcellular penetration of the blood-brain barrier. Infect Immun. 2004;72(9):4985–95. doi: 10.1128/IAI.72.9.4985-4995.2004 15321990 PMC517459

[4] Na Pombejra S, Salemi M, Phinney BS, Gelli A. The metalloprotease, Mpr1, engages AnnexinA2 to promote the transcytosis of fungal cells across the blood-brain barrier. Front Cell Infect Microbiol. 2017;7:296. doi: 10.3389/fcimb.2017.00296 28713781 PMC5492700

[5] Rajasingham R, Govender NP, Jordan A, Loyse A, Shroufi A, Denning DW, et al. The global burden of HIV-associated cryptococcal infection in adults in 2020: a modelling analysis. Lancet Infect Dis. 2022;22(12):1748–55. doi: 10.1016/S1473-3099(22)00499-6 36049486 PMC9701154

[6] Chen SHM, Stins MF, Huang S-H, Chen YH, Kwon-Chung KJ, Chang Y, et al. Cryptococcus neoformans induces alterations in the cytoskeleton of human brain microvascular endothelial cells. J Med Microbiol. 2003;52(Pt 11):961–70. doi: 10.1099/jmm.0.05230-0 14532340

[7] Vu K, Tham R, Uhrig JP, Thompson GR 3rd, Na Pombejra S, Jamklang M, et al. Invasion of the central nervous system by Cryptococcus neoformans requires a secreted fungal metalloprotease. mBio. 2014;5(3):e01101-14. doi: 10.1128/mBio.01101-14 24895304 PMC4049100

[8] Na Pombejra S, Jamklang M, Uhrig JP, Vu K, Gelli A. The structure-function analysis of the Mpr1 metalloprotease determinants of activity during migration of fungal cells across the blood-brain barrier. PLoS One. 2018;13(8):e0203020. doi: 10.1371/journal.pone.0203020 30161190 PMC6117016

[9] Aaron P, Gelli A. Harnessing the activity of the fungal metalloprotease, Mpr1, to promote crossing of nanocarriers through the blood-brain barrier. ACS Infect Dis. 2019. 10.1021/acsinfecdis.9b00348 31820926

[10] Nielsen K, Cox GM, Wang P, Toffaletti DL, Perfect JR, Heitman J. Sexual cycle of Cryptococcus neoformans var. grubii and virulence of congenic a and alpha isolates. Infect Immun. 2003;71(9):4831–41. doi: 10.1128/IAI.71.9.4831-4841.2003 12933823 PMC187335

[11] Weksler BB, Subileau EA, Perrière N, Charneau P, Holloway K, Leveque M, et al. Blood-brain barrier-specific properties of a human adult brain endothelial cell line. FASEB J. 2005;19(13):1872–4. doi: 10.1096/fj.04-3458fje 16141364

[12] Turner A, Lanser DM, Gelli A. Optimized expression and isolation of recombinant active secreted proteases using Pichia pastoris. Bio Protoc. 2023;13(5):e4628. 10.21769/BioProtoc.4628 PMC9993078 36908634

[13] McCall RL, Sirianni RW. PLGA nanoparticles formed by single- or double-emulsion with vitamin E-TPGS. J Vis Exp. 2013;82:51015. 10.3791/51015 PMC4106449 24429733

[14] Allen R, Chizari S, Ma JA, Raychaudhuri S, Lewis JS. Combinatorial, microparticle-based delivery of immune modulators reprograms the dendritic cell phenotype and promotes remission of collagen-induced arthritis in mice. ACS Appl Bio Mater. 2019;2(6):2388–404. doi: 10.1021/acsabm.9b00092 35030696

[15] Sangsuwan R, Yik JHN, Owen M, Liu G-Y, Haudenschild DR, Lewis JS. Intra-articular injection of flavopiridol-loaded microparticles for treatment of post-traumatic osteoarthritis. Acta Biomater. 2022;149:347–58. doi: 10.1016/j.actbio.2022.06.042 35779774 PMC10281459

[16] Lewis JS, Stewart JM, Marshall GP, Carstens MR, Zhang Y, Dolgova NV, et al. Dual-sized microparticle system for generating suppressive dendritic cells prevents and reverses type 1 diabetes in the nonobese diabetic mouse model. ACS Biomater Sci Eng. 2019;5(5):2631–46. doi: 10.1021/acsbiomaterials.9b00332 31119191 PMC6518351

[17] Hamill RJ. Amphotericin B formulations: a comparative review of efficacy and toxicity. Drugs. 2013;73(9):919–34. doi: 10.1007/s40265-013-0069-4 23729001

[18] Amaral AC, Bocca AL, Ribeiro AM, Nunes J, Peixoto DLG, Simioni AR, et al. Amphotericin B in poly(lactic-co-glycolic acid) (PLGA) and dimercaptosuccinic acid (DMSA) nanoparticles against paracoccidioidomycosis. J Antimicrob Chemother. 2009;63(3):526–33. doi: 10.1093/jac/dkn539 19151037

[19] Vu K, Weksler B, Romero I, Couraud P-O, Gelli A. Immortalized human brain endothelial cell line HCMEC/D3 as a model of the blood-brain barrier facilitates in vitro studies of central nervous system infection by Cryptococcus neoformans. Eukaryot Cell. 2009;8(11):1803–7. doi: 10.1128/EC.00240-09 19767445 PMC2772405

[20] Poller B, Gutmann H, Krähenbühl S, Weksler B, Romero I, Couraud P-O, et al. The human brain endothelial cell line hCMEC/D3 as a human blood-brain barrier model for drug transport studies. J Neurochem. 2008;107(5):1358–68. doi: 10.1111/j.1471-4159.2008.05730.x 19013850

[21] Hines DJ, Kaplan DL. Poly(lactic-co-glycolic) acid-controlled-release systems: experimental and modeling insights. Crit Rev Ther Drug Carrier Syst. 2013;30(3):257–76. doi: 10.1615/critrevtherdrugcarriersyst.2013006475 23614648 PMC3719420

[22] Zhang W, Mehta A, Tong Z, Esser L, Voelcker NH. Development of polymeric nanoparticles for blood-brain barrier transfer-strategies and challenges. Adv Sci (Weinh). 2021;8(10):2003937. doi: 10.1002/advs.202003937 34026447 PMC8132167

[23] Lombardo SM, Schneider M, Türeli AE, Günday Türeli N. Key for crossing the BBB with nanoparticles: the rational design. Beilstein J Nanotechnol. 2020;11:866–83. doi: 10.3762/bjnano.11.72 32551212 PMC7277618

[24] Van de Ven H, Paulussen C, Feijens PB, Matheeussen A, Rombaut P, Kayaert P, et al. PLGA nanoparticles and nanosuspensions with amphotericin B: potent in vitro and in vivo alternatives to Fungizone and AmBisome. J Control Release. 2012;161(3):795–803. doi: 10.1016/j.jconrel.2012.05.037 22641062

[25] Saraiva C, Praça C, Ferreira R, Santos T, Ferreira L, Bernardino L. Nanoparticle-mediated brain drug delivery: overcoming blood-brain barrier to treat neurodegenerative diseases. J Control Release. 2016;235:34–47. doi: 10.1016/j.jconrel.2016.05.044 27208862

[26] Yang M, Du K, Hou Y, Xie S, Dong Y, Li D, et al. Synergistic antifungal effect of amphotericin B-loaded poly(lactic-co-glycolic acid) nanoparticles and ultrasound against Candida albicans biofilms. Antimicrob Agents Chemother. 2019;63(4). 10.1128/AAC.02022-18 PMC6437511 30670414

[27] Maji A, Soutar CP, Zhang J, Lewandowska A, Uno BE, Yan S, et al. Tuning sterol extraction kinetics yields a renal-sparing polyene antifungal. Nature. 2023;623(7989):1079–85. doi: 10.1038/s41586-023-06710-4 37938782 PMC10883201

[28] Aaron PA, Vu K, Gelli A. An antivirulence approach for preventing Cryptococcus neoformans from crossing the blood-brain barrier via novel natural product inhibitors of a fungal metalloprotease. mBio. 2020;11(4):e01249-20. doi: 10.1128/mBio.01249-20 32694141 PMC7374060

[29] Chang J, Jallouli Y, Kroubi M, Yuan X, Feng W, Kang C, et al. Characterization of endocytosis of transferrin-coated PLGA nanoparticles by the blood-brain barrier. Int J Pharm. 2009;379(2):285–92. doi: 10.1016/j.ijpharm.2009.04.035 19416749

[30] Sharma S, Tyagi A, Dang S. Nose to brain delivery of transferrin conjugated PLGA nanoparticles for clonidine. Int J Biol Macromol. 2023;252:126471. doi: 10.1016/j.ijbiomac.2023.126471 37619678

[31] Lu W, Wan J, She Z, Jiang X. Brain delivery property and accelerated blood clearance of cationic albumin conjugated pegylated nanoparticle. J Control Release. 2007;118(1):38–53. doi: 10.1016/j.jconrel.2006.11.015 17240471

[32] Lin T, Zhao P, Jiang Y, Tang Y, Jin H, Pan Z, et al. ACS Nano. 2016;10(11):9999–10012. 27934069 10.1021/acsnano.6b04268

[33] Abbott NJ, Rönnbäck L, Hansson E. Astrocyte-endothelial interactions at the blood-brain barrier. Nat Rev Neurosci. 2006;7(1):41–53. doi: 10.1038/nrn1824 16371949

[34] Ranaivo HR, Wainwright MS. Albumin activates astrocytes and microglia through mitogen-activated protein kinase pathways. Brain Res. 2010;8:1313-222–31. 10.1016/j.brainres.2009.11.063 PMC2812578 19961838

[35] Teleanu RI, Preda MD, Niculescu A-G, Vladâcenco O, Radu CI, Grumezescu AM, et al. Current strategies to enhance delivery of drugs across the blood-brain barrier. Pharmaceutics. 2022;14(5):987. doi: 10.3390/pharmaceutics14050987 35631573 PMC9145636

[36] Yuen CM, Liu DR. Dissecting protein structure and function using directed evolution. Nat Methods. 2007;4(12):995–7. doi: 10.1038/nmeth1207-995 18049466 PMC4255555

