# Supplementary figures and images for: Conjugation of a Cryptococcus neoformans-derived metalloprotease to antifungal-loaded PLGA nanoparticles treats neural cryptococcosis in an in vitro model
Source: PLoS One. 2026 Jan 16;21(1):e0340202. doi: 10.1371/journal.pone.0340202 (PMC12810805; doi:10.1371/journal.pone.0340202)

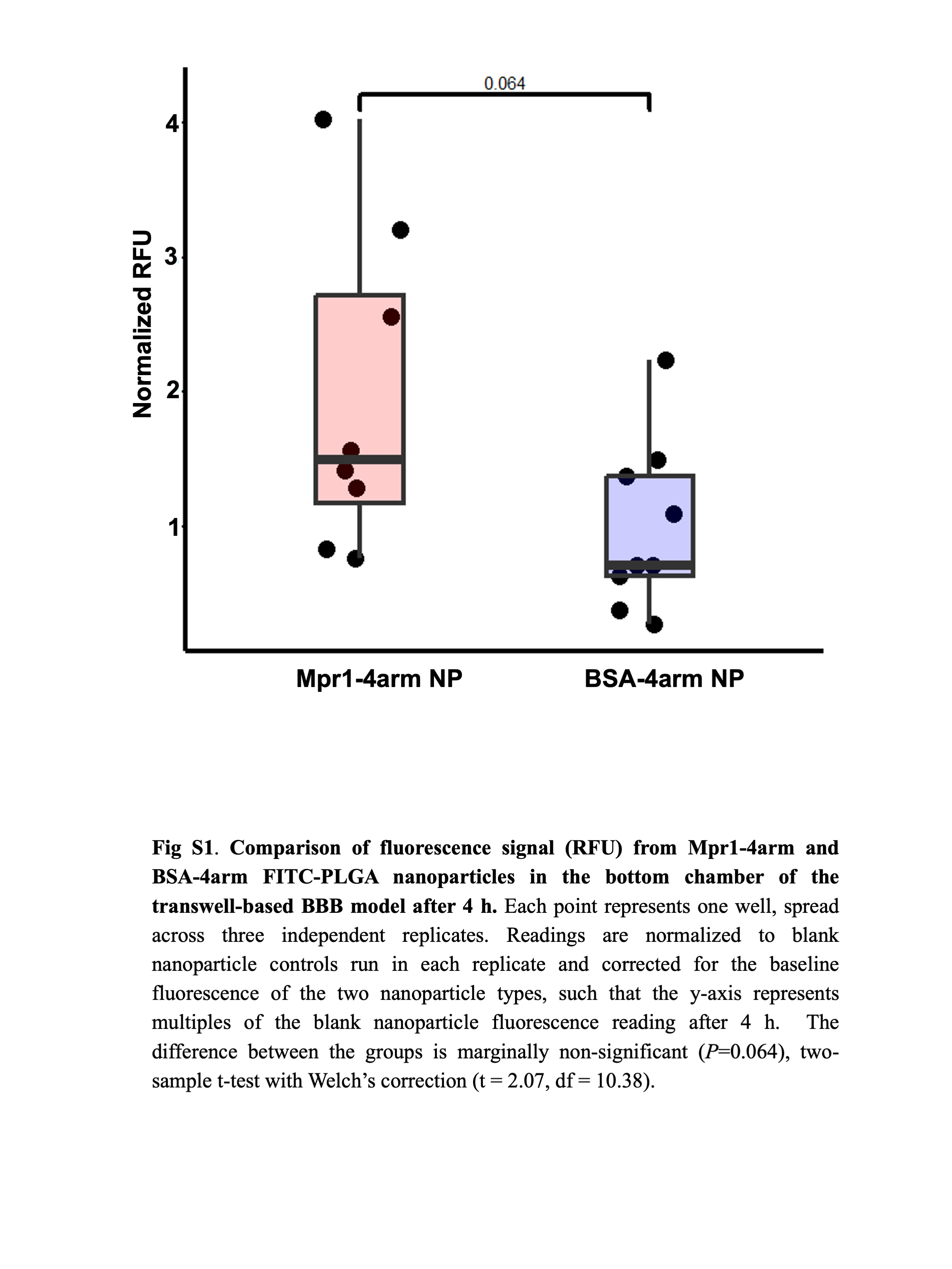

Supplement: S1 Fig — Each point represents one well, spread across three independent replicates. Readings are normalized to blank nanoparticle controls run in each replicate and corrected for the baseline fluorescence of the two nanoparticle types, such that the y-axis represents multiples of the blank nanoparticle fluorescence reading after 4 h. The difference between the groups is marginally non-significant (P = 0.064), two-sample t-test with Welch’s correction (t = 2.07, df = 10.38). (TIFF) [file pone.0340202.s001.tiff]
